# Dentists’ Perspective on the Impact of COVID-19 on the Utilization of Emergency Dental Services in Kuwait: A Cross-Sectional Study

**DOI:** 10.3390/clinpract13030058

**Published:** 2023-05-18

**Authors:** Nour Alonaizi, Sarah Alharran, Jagan Kumar Baskaradoss

**Affiliations:** 1 Department of General Dentistry, Ministry of Health, Kuwait City 13001, Kuwait; 2 Department of Developmental and Preventive Sciences, College of Dentistry, Kuwait University, Kuwait City 13110, Kuwait

**Keywords:** COVID-19, dental service, perception, utilization

## Abstract

This cross-sectional study intends to evaluate dentists’ perceptions of COVID-19’s effects on the use of emergency dental care both during and after Kuwait’s lockdown periods. A convenience sample of dentists employed by the Ministry of Health’s various emergency dental clinics and School Oral Health Programs (SOHP) throughout Kuwait’s six governorates were invited to take part in this study. To determine the impact of various demographic and occupational characteristics on the mean perception score of the dentist, a multi-variable model was developed. The study was conducted between June–September 2021, and a total of 268 dentists (61% males and 39% females) participated in this study. When compared to pre-lockdown periods, the overall number of patients seen by dentists had significantly decreased after the lockdown period. After lockdown, there were significantly more cases of acute pulpitis with apical periodontitis, abscesses, and pericoronitis than there were before lockdown (*p* < 0.05). After the lockdown, a significantly higher percentage of dentists (*p* < 0.05) reported using fewer droplets-generating procedures to manage patients with dental emergencies. After correcting for the other variables in the model, female dentists (β = 0.146; 95% CI = 0.071 to 1.451) and non-Kuwaiti dentist (β = 0.012; 95% CI = 0.234 to 1.854) had a significantly (*p* < 0.05) more positive perception of the utilization of dental services than others after adjusting for the other variables in the model. The majority of dentists perceive that the COVID-19 pandemic has had a negative impact on Kuwait’s use of emergency dental services.

## 1. Introduction

The global spread of the coronavirus disease (COVID-19) has put a strain on the world’s healthcare systems that has never been seen before. Globally, 6.7 million COVID-19 deaths have been documented, along with more than 671 million confirmed cases [1]. The first reported case of COVID-19 in Kuwait occurred in the last week of February 2020 [2]. The Ministry of Health, Kuwait (MoH) recorded 2570 fatalities out of about 663,000 confirmed COVID-19 cases [3].

The Kuwaiti government implemented rules and restrictions to curb the spread of the virus as the number of cases rose, including a ban on gatherings and parties; the closure of schools, universities, and mosques; as well as the institution of curfews. Dental treatments given in the public and private sectors were subjected to restrictions. Dentists were categorized as being in a “very-high-risk” group [4] based on their line of practice. Soon after the pandemic began, the American Dental Association (ADA) recommended that dental clinics suspend all routine or elective treatment and manage only dental emergencies and urgent care in order to reduce the risk of spread of infection. Following this, the Ministry of Health, Kuwait, limited dental care to emergency situations from March to July 2020 [5]. Emergency dental procedures were listed by several dental associations including the ADA [6] and the Royal College of Surgeons, England [7]. In brief, dental emergency procedures would include but not be limited to the following situations [8,9]: (1) dental trauma (which needs intervention and treatment under local anesthetic or sedation is not possible); (2) acute dental infection (that is not responsive to antibiotics); (3) intractable pain (which cannot be managed under local anesthetic); (4) facial swelling as a result of dental disease (where treatment under local anesthetic is not possible); (5) poor dental health compromising general health. The performance of all non-emergency procedures and treatments that generate aerosols or droplets was required to wait until after the lockdown.

Health-seeking behaviors reportedly tend to alter during pandemics, according to several research studies [10,11,12]. During pandemics, self-medication has been found to increase, while healthcare services are underutilized [11]. Similar to this, most patients with acute respiratory infections during the COVID-19 pandemic chose not to seek medical attention [13]. Dental services were used differently during COVID-19, according to several studies [14,15,16,17]. Emergency dental visits were shown to be on the rise in certain studies [14,15,18,19], whereas others [20] found no discernible increase in these visits. Information on how the COVID-19 pandemic has affected the use of dental emergency care is scarce. Overall, the COVID-19 pandemic has led to a significant reduction in the utilization of dental services, which could have negative long-term effects on oral health. It is essential for dental professionals and policymakers to find ways to provide dental services safely and effectively to ensure that people continue to receive necessary care. This is the first study assessing emergency dental clinic utilization in this population. This study intends to investigate how dentists perceive COVID-19’s impact on Kuwait’s emergency dental treatment utilization patterns.

## 2. Materials and Methods

### 2.1. Study Design and Sampling

The Ethics Committee of Kuwait University at the Health Science Centre (HSC) approved this study (VDR/EC/258; dated 2 February 2021). Permission to conduct the study in the selected hospitals was obtained from the Ministry of Health and from the concerned authorities at the selected hospitals. STrengthening the Reporting of OBservational studies in Epidemiology (STROBE) guidelines have been followed in the preparation of this manuscript [21].

Data collection was performed between June–September 2021. There are six administrative blocks or governorates in Kuwait. A list of all the tertiary care hospitals and dental clinics providing dental emergency services in the six governorates were obtained from the Ministry of Health. In this cross-sectional study, all dentists who worked in the emergency dental services at the selected hospitals were invited to participate in this study. All the participants signed the written informed consent form prior to participating in this study.

Dentists employed by government dental clinics, specialty dentistry centers, and school oral health programs were given a questionnaire (SOHP). From each of Kuwait’s six governorates, one governmental specialist dentistry center and one SOHP dental clinic were chosen based on convenience (total centers: 12). Also, dentists employed at Kuwait University Dental Centre (KUDC) were invited to take part in the study.

### 2.2. Survey Instrument

There were 21 questions under four domains in the questionnaire. Multiple-choice questions about the demographic characteristics of dentists make up the first section. The second section covered reported cases before and after lockdown as well as emergency dental services. The third section covers the patient care provided and the preventative measures taken both before and after the lockdown. The last part was about dentist opinion about protective measurement, awareness, and impact of COVID-19 on their lives.

### 2.3. Statistical Analysis

Sample size estimation was performed using the G* Power software package (version 3.1.9.6). For an assumed effect size of 0.3, with an alpha error of 0.05 and 5 degrees of freedom, a sample of 250 respondents would result in a power of 76%. The final sample size was set at 300 subjects in order to account for the missing data. Chi-square and Fisher’s exact tests were used to test the significance of associations for categorical variables. A multivariable model assessing the influence of the various demographic and work-related factors on the mean perception score of the dentist was built using the enter method. The level of significance was set to *p* < 0.05. All bivariate and multivariable analyses were carried out using SPSS 27.0 (IBM Corp. Released 2017. IBM SPSS Statistics for Windows, Version 27.0. Armonk, NY, USA: IBM Corp.).

## 3. Results

### Dentist Characteristics

A total of 268 dentists (61% males and 39% females) participated in this study, and their demographic characteristics stratified by years of experience (<10 years and ≥10 years) are listed in Table 1. Most of the respondents were Kuwaiti nationals (60%) younger than 40 years old (70%), and about 43% of the respondents had less than 10 years’ clinical experience. As compared with dentists who had 10 or more years of experience, dentists with less than 10 years’ experience had a higher proportion of general dental practitioners (GDPs) with only a bachelor’s degree.

Table 2 presents the dentists’ perception of the utilization pattern of emergency dental services before and after the lockdown period due to the COVID-19 pandemic. The total number of patients seen by dentists had significantly reduced after lockdown compared with before lockdown (*p* < 0.001). Pain was the most common reason for emergency dental visits both before and after lockdown. The most frequent chief complaint was pain alone (32.3%), followed by pain and swelling (15.5%). The most frequent principal diagnosis was acute pulpitis (33.2%), followed by acute pulpitis with apical periodontitis (11.9%). The dentists’ perception of the primary chief complaints for seeking emergency dental treatment were significantly different before and after the COVID-19 lockdown (*p* < 0.001). A significantly higher number of dentists reported attending to patients with pain and swelling after lockdown as compared with before lockdown (*p* < 0.001). The causes of tooth ache were also significantly different before and after lockdown. A higher proportion of dentists reported patients presenting with acute pulpitis with apical periodontitis and abscesses with pericoronitis after lockdown as compared with before lockdown (*p* < 0.001).

The dentists reported significant differences in the management of dental emergencies before and after lockdown (Table 3). A significantly higher proportion of dentists reported being afraid of contracting COVID-19 after the lockdown period as compared with before lockdown. The practices of recording patients’ temperatures, use of personal protective equipment (PPE), and asking patients to use mouthwash prior to procedures had all significantly increased after lockdown as compared with before (*p* < 0.001).

Table 4 presents the multivariable linear regression model on the influence of selected demographic and work-related factors on the perception of dentists. Female (β = 0.146; 95% CI = 0.071 to 1.451) and non-Kuwaiti dentists (β = 0.012; 95% CI = 0.234 to 1.854) had a significantly (*p* < 0.05) more positive perception of the utilization of dental services than others after adjusting for the other variables in the model.

## 4. Discussion

In the study, emergency dental services in Kuwait were compared before and after the lockdown periods using a retrospective descriptive analysis. The findings imply that COVID-19 had a considerable impact on dentists’ perceptions of Kuwait’s emergency dental treatment consumption patterns.

In this study, the dentists report seeing fewer patients at the emergency room following the COVID-19 lockdown period. Several studies [20,22] also observed findings that were similar. According to a Taiwanese study, during a lockdown, 17% fewer patients visited dental emergency rooms [20]. According to Hahn et al. [22], there was a 50% decrease in dental emergency patients after the lockdown. Fear of contracting an illness when undergoing dental work may have decreased the use of emergency dental services.

Dental pain and pain with swelling were the two main complaints among emergency dentistry patients, and they differed noticeably before and after the COVID-19 lockdown periods. This result was at odds with the findings of several other studies. Trauma was the most frequent diagnosis in emergency rooms both before and after the COVID-19 outbreak, according to research by Wu et al. [20]. Guo et al. [14] discovered that dental emergency visits in China were primarily brought on for non-traumatic reasons (dental pulpal or periapical lesions). According to a study from the USA [23], dental caries and pulpitis or periapical abscess are the top reasons people need to see an emergency dentist. The most frequent causes of patient visits to the emergency room in France were cellulitis or abscesses and dental pulpal or periapical lesions [24]. The various perspectives of the population, current health department laws, and accessibility to dental care facilities could all contribute to the variations in the causes of emergency dental visits. Also, the curfew’s widespread adoption and a decline in outdoor activities may have helped to lower the number of trauma cases. Delays in seeking dental care may have been caused by anxiety about catching COVID-19 during dental procedures.

Before and after the lockdown, dentists’ perceptions of the use of emergency dental care varied greatly. After the lockdown period, a greater percentage of dentists than before the lockdown reported being fearful of contracting COVID-19.

The number of dentists providing dental services that produced droplets and aerosols significantly reduced after the lockdown. There was an increase in the emphasis placed on dental triage after lockdown. The recording of patients’ temperatures, use of PPE, and asking to use mouthwash all significantly increased after lockdown as compared with before. The fear of contracting COVID-19 during dental treatment could have contributed to the change in practice patterns.

Female dentists and non-Kuwaiti dentists had significantly better perception scores than others after adjusting for the other variables in the multivariable model. Gender differences in knowledge and practice between male and female dentists have been previously reported in the literature [25]. A higher proportion of male dentists participated in this study compared with female; however, female dentists had significantly better perception scores. Most of the respondents were Kuwaiti dentists; however, non-Kuwaiti dentists had better perception scores than Kuwaiti dentists.

The majority of respondents to this survey believed that the published COVID-19 recommendations could be safely administered if they were followed, and that they should be updated often. There was little cause for concern among the respondents about practicing dentists acquiring COVID-19. Additionally, they believe that it is important to follow the recommended treatment protocols even while providing basic care. The majority of respondents thought that following recommended infection control methods is necessary in order to maintain an infection-free workplace.

The study’s shortcomings should be taken into account when interpreting the results. First off, since this was a cross-sectional study, we are unable to draw any conclusions about causality. Additionally, because the study’s findings were based exclusively on a questionnaire survey, cross-validation of the data using official treatment records was not possible. Lastly, a convenient sampling technique was adopted for this study, which limits the generalizability of the study’s findings.

## 5. Conclusions

To conclude, the spread of the COVID-19 pandemic affected the utilization pattern of emergency dental services in Kuwait. The number of visits to dental emergency facilities significantly reduced during COVID-19, and pain was the most common principal reason for seeking dental care. Dental healthcare must be carefully planned and managed in order to adjust to the new circumstances.

## Figures and Tables

**Table 1:** Socio-demographic characteristics stratified by years of experience.

Variables	Total *n* (%)	Years of Experience		
		<10 Years *n* (%)	≥10 Years *n* (%)	*p*-Value *
**Total**	268 (100.0)	117 (43.7)	151 (56.3)	0.116
Dental centers				
Al-Amiri	45 (16.8)	19 (16.2)	26 (17.2)	
Al-Adan	33 (12.3)	12 (10.3)	21 (13.9)	
Jahra	46 (17.2)	15 (12.8)	31 (20.5)	
Farwaniya	12 (4.5)	4 (3.4)	8 (5.3)	
Jaber	37 (13.8)	20 (17.1)	17 (11.3)	
Bneid Al Gar	37 (13.8)	13 (11.1)	24 (15.9)	
SOHP	43 (16.0)	25 (21.4)	18 (11.9)	
Others	15 (5.6)	9 (7.7)	6 (4.0)	
Gender				0.030
Male	164 (61.2)	63 (53.8)	101 (66.9)	
Female	104 (38.8)	54 (46.2)	50 (33.1)	
Age				<0.001
21–40	187 (69.8)	114 (97.4)	73 (48.3)	
41–60	68 (25.4)	3 (2.6)	65 (43)	
≥60	13 (4.9)	0 (0.0)	13 (8.6)	
Nationality				0.002
Kuwaiti	162 (60.4)	83 (70.9)	79 (52.3)	
Non-Kuwaiti	106 (39.6)	34 (29.1)	72 (47.7)	
Marital status				<0.001
Single	75 (28.0)	59 (50.4)	16 (10.6)	
Married	184 (68.7)	56 (47.9)	128 (84.8)	
Divorced	9 (3.4)	2 (1.7)	7 (4.6)	
Number of children				<0.001
<3	109 (40.7)	34 (29.1)	75 (49.7)	
3 or more	64 (23.9)	9 (7.7)	55 (36.4)	
No children	95 (35.4)	74 (63.2)	21 (13.9)	
Level of education				<0.001
Bachelor	86 (32.1)	64 (54.7)	22 (14.6)	
Master	89 (33.2)	31 (26.5)	58 (38.4)	
Board/PhD	93 (34.7)	22 (18.8)	71 (47.0)	
Field of practice				<0.001
General dentist	64 (23.9)	54 (46.2)	10 (6.6)	
Pediatric dentistry	47 (17.5)	17 (14.5)	30 (19.9)	
Prosthodontics	30 (11.2)	10 (8.5)	20 (13.2)	
Orthodontics	31 (11.6)	10 (8.5)	21 (13.9)	
Periodontics	21 (7.8)	6 (5.1)	15 (9.9)	
Surgery	33 (12.3)	9 (7.7)	24 (15.9)	
Endodontics	30 (11.2)	9 (7.7)	21 (13.9)	
Others	12 (4.5)	2 (1.7)	10 (6.6)	

* Chi-square or Fisher’s exact tests. SOHP- School Oral Health Program.

**Table 2:** Dentists’ perception of the utilization pattern of emergency dental services before and after the lockdown period due to the COVID-19 pandemic.

Variables	Total *n* (%)	Before Lockdown *n* (%)	After Lockdown *n* (%)	*p*-Value
**How many dental emergency patients do you attend to in a day?**				
1–2	92 (17.2)	49 (18.3)	43 (16.0)	<0.001
3–4	160 (29.9)	73 (27.2)	87 (32.5)	
≥5	269 (50.2)	140 (52.2)	129 (48.1)	
**What is the most common age group of emergency patients you see?**				
<18 years old	206 (38.4)	99 (36.9)	107 (39.9)	<0.001
18–40 years old	196 (36.6)	102 (38.1)	94 (35.1)	
40–65 years old	41 (7.7)	20 (7.5)	21 (7.8)	
>65 years old	4 (0.8)	1 (0.4)	3 (1.1)	
All age groups	51 (9.6)	27 (10.1)	24 (9.0)	
>40 years old	30 (5.6)	16 (6)	14 (5.2)	
**What is the most common chief complaints of the patients you see?**				
Trauma	21 (3.9)	13 (4.9)	8 (3.0)	<0.001
Pain	173 (32.3)	90 (33.6)	83 (31)	
Bleeding	10 (1.9)	8 (3.0)	2 (0.7)	
Swelling	6 (1.2)	1 (0.4)	5 (1.9)	
Pain, Swelling and Bleeding	80 (14.9)	40 (14.9)	40 (14.9)	
Pain and bleeding	27 (5.0)	10 (3.7)	17 (6.3)	
Pain and swelling	83 (15.5)	34 (12.7)	49 (18.3)	
Trauma, pain & swelling	53 (9.9)	32 (11.9)	21 (7.8)	
Others	72 (13.4)	36 (13.4)	36 (13.4)	
**What were the most common dental diagnosis made at the emergency clinics for acute tooth ache?**				
Acute pulpitis	178 (33.2)	97 (36.2)	81 (30.2)	<0.001
Acute periapical periodontitis	38 (7.1)	16 (6.0)	22 (8.2)	
Combined periodontic endodontic lesions	19 (3.6)	9 (3.4)	10 (3.7)	
Periodontitis	26 (4.9)	12 (4.5)	14 (5.2)	
Acute pulpitis, acute periapical periodontitis and combined periodontic endodontic lesions	60 (11.2)	31 (11.6)	29 (10.8)	
Pulpitis and periodontitis	56 (10.5)	30 (11.2)	26 (9.7)	
Acute pulpitis and apical periodontitis	64 (11.9)	28 (10.4)	36 (13.4)	
Others	34 (6.3)	17 (6.3)	17 (6.3)	
**What were the most common dental diagnosis made at the emergency clinics for maxillofacial and dental trauma?**				
Dental trauma	190 (35.5)	96 (35.8)	94 (35.1)	<0.001
Soft tissue injury	42 (7.9)	24 (9.0)	18 (6.7)	
Jaw fracture	25 (4.7)	9 (3.4)	16 (6)	
Trauma, soft tissue injury and jaw fracture	25 (4.7)	15 (5.6)	10 (3.7)	
Others	26 (4.9)	13 (4.9)	13 (4.9)	
Maxillofacial trauma	42 (7.9)	20 (7.5)	22 (8.2)	
**What were the most common dental diagnosis made at the emergency clinics for dental infections?**				
Abscess	229 (42.8)	120 (44.8)	109 (40.7)	<0.001
Pericoronitis	50 (9.35)	27 (10.1)	23 (8.6)	
Maxillofacial space infection	19 (3.6)	3 (1.1)	16 (6.0)	
Others	21 (3.9)	10 (3.7)	11 (4.1)	
All the above	50 (9.4)	26 (9.7)	24 (9.0)	

**Table 3:** Differences in the practice of managing dental emergencies before and after lockdown.

Variables	Total	Lockdown		*p*-Value
	*n* (%)	Before	After	
How were the emergency cases managed?				
Using dental procedures that do not produce droplets	55 (10.3)	20 (7.5)	35 (13.1)	<0.001
Using dental procedures that produce droplets	177 (33.0)	110 (41.0)	67 (25.0)	
Prescribing Antibiotics only	32 (5.9)	15 (5.6)	17 (6.3)	
Prescribing antibiotics with non-aerosol generating procedures	246 (45.9)	114 (42.5)	132 (49.3)	
You were afraid of being infected with COVID-19 from your patients?				
Always	240 (44.8)	92 (34.3)	148 (55.2)	<0.001
Rarely	189 (35.3)	116 (43.3)	73 (27.2)	
Never	101 (18.8)	58 (21.6)	43 (16)	
You tried to avoid performing high risk procedures				
Always	115 (21.5)	40 (14.9)	75 (28.0)	<0.001
Rarely	191 (35.6)	92 (34.3)	99 (36.9)	
Never	225 (42.0)	135 (50.4)	90 (33.6)	
You record patient temperature				
Always	250 (46.7)	78 (29.1)	172 (64.2)	<0.001
Rarely	98 (18.3)	56 (20.9)	42 (15.7)	
Never	176 (32.8)	128 (47.8)	48 (17.9)	
You use full PPE before attending to your patients				
Always	374 (69.8)	154 (57.5)	220 (82.1)	<0.001
Rarely	112 (20.9)	78 (29.1)	34 (12.7)	
Never	46 (8.6)	35 (13.1)	11 (4.1)	
You ask patient to use mouthwash before treatment				
Always	161 (30.1)	72 (26.9)	89 (33.2)	<0.001
Rarely	192 (35.9)	98 (36.6)	94 (35.1)	
Never	171 (31.9)	93 (34.7)	78 (29.1)	

**Table 4:** Multivariable linear regression model for demographic and work-related factors that influences the perception of dentists.

	Unstandardized Coefficients		Standardized Coefficients	*p*-Value	95.0% Confidence Interval for B	
	B	Std. Error	Beta		Lower Bound	Upper Bound
(Constant)	6.655	1.332		<0.001	4.031	9.28
Dental center	−0.044	0.051	−0.058	0.392	−0.144	0.057
Gender	0.761	0.35	0.146	0.031	0.071	1.451
Age group	0.256	0.469	0.057	0.585	−0.667	1.18
Nationality	1.044	0.411	0.201	0.012	0.234	1.854
Educational status	0.121	0.271	0.039	0.657	−0.413	0.654
Specialty	−0.011	0.072	−0.011	0.883	−0.153	0.132
Years of work experience	−0.361	0.329	−0.119	0.274	−1.009	0.287
Received covid vaccine	−1.001	0.741	−0.088	0.178	−2.461	0.458

Dependent Variable: Perception score.

## Data Availability

The data used in this study are available from the corresponding author on reasonable request.
